# The influence of permeability and heterogeneity on chemical flooding efficiency and remaining oil distribution—based on NMR displacement imaging

**DOI:** 10.1038/s41598-023-39535-2

**Published:** 2023-08-31

**Authors:** Yang Zhang, Changcheng Gai, Binghui Song, Jiguo Jiang, Zhiqiang Wang

**Affiliations:** 1 Karamay Campus, Faculty of Petroleum, China University of Petroleum (Beijing), Karamay, 834000 Xinjiang China; 2 Research Institute of Exploration and Development, PetroChina Jidong Oilfield Company, Tangshan, 063004 Hebei China; 3 Research Institute of Exploration and Development, PetroChina Tarim Oilfield Company, Korla, 841000 Xinjiang China; 4 Second Oil Production Plant, PetroChina Xinjiang Oilfield Company, Karamay, 834000 Xinjiang China

**Keywords:** Geology, Petrology

## Abstract

To investigate the impact of permeability and heterogeneity on oil displacement efficiency and remaining oil distribution of chemical flooding, three groups of high and ultrahigh permeability core samples from an ultrahigh water-cut oilfield in western China were selected as the research objects in this study. High-pressure mercury injection, scanning electron microscopy, wettability test, and other methods were used to characterize the reservoir properties of core samples. Six groups of experiments were performed using the nuclear magnetic resonance (NMR) displacement imaging technology to simulate the oilfield development process considering the economic benefits. The displacement stage with the best oil displacement effect in the process of waterflooding, chemical flooding and then waterflooding was defined, and the control effect of permeability and heterogeneity on the improvement of oil displacement efficiency by polymer-surfactant binary flooding was discussed. The distribution position of remaining oil in different displacement stages was quantitatively and visually displayed, and its control factors were revealed. The research shows that during the simulation process of first waterflooding followed by chemical flooding and then waterflooding in the oilfield, the T_2_ spectrum signal amplitude increases the most in the two stages, one is from saturated oil flooding to 50% water cut and the other one is from 95% water cut to the end of 1 PV polymer flooding. The oil displacement efficiency increases the most, and the oil is primarily discharged from pore throats larger than 90 ms (or with pore throat radius of 8.37 μm). Compared with heterogeneity, permeability plays a more obvious controlling role in improving the oil displacement efficiency of polymer-surfactant binary flooding. The influence of fingering phenomenon on the distribution of remaining oil is most obvious in the second waterflooding, and the distribution of remaining oil with polymer slug is more obviously affected by the fingering phenomenon than that with polymer-surfactant slug. The study results provide theoretical guidance for tapping the remaining oil potential of old oilfields with high to ultrahigh permeabilities.

## Introduction

At the high water-cut period in the later stage of oilfield development, a large amount of remaining oil remains underground after waterflooding^1^. Under current economic and technical conditions, 25–35% of the oil and gas remain underground due to the influence of internal heterogeneity within the reservoir and become movable macro rem oil. By adjusting the waterflooding injection-production well pattern and fine separate injection, this portion of the oil and gas can be further displaced and discharged. Furthermore, 30%–40% of the oil and gas cannot be discharged because the water does not penetrate or is affected by interfacial tension, capillary force, and other resistances. They are distributed in the reservoir pores and throats as accumulated oil spots or dispersed oil droplets, and conventional removal methods, such as waterflooding, are ineffective. As a result, a variety of tertiary oil recovery techniques have been developed^2–4^.

Chemical flooding is a type of tertiary oil recovery method that can increase sweep efficiency and improve oil displacement efficiency by introducing a chemical agent into the injected water, thus changing the physical and chemical properties of the displacement fluid as well as the interfacial properties between the displacement fluid and the crude oil and rock minerals, mainly including polymer flooding^5,6^, polymer/surfactant binary flooding^7^, and surfactant/polymer/alkali ternary flooding^8,9^. Chemical flooding has become an important tool in China for significantly increasing oil recovery in medium- and high-permeability oilfields during the late development period^1^. Previous research has shown that polymer flooding can reduce the water/oil mobility ratio, increase the conformance efficiency of injected water in the reservoir, and improve the micro-oil washing efficiency^10^. The surfactant flooding is relatively high-cost, but it can effectively reduce the oil–water interfacial tension^11,12^. Suitable Surfactant can change the surface of rock from lipophilicity to hydrophilicity, and make capillary force to become the driving force, reduce the adhesion of rock surface and improve the oil washing efficiency^13^. The binary and ASP flooding processes generate ultralow interfacial tension and emulsification, they can enhance oil recovery significantly by fully tapping the remaining oil in small and medium pores and on the surface of pores^10^. However, some side effects have to be considered, with the migration of the composite system solution to the production well, the formation sandstone has a retention and loss effect on the polymer and surfactant, which can lead to the risk of a severe polymer induced permeability reduction^14–16^. The economic input of binary and ASP flooding depends on many factors such as chemical agent cost, operation cost, surface construction cost and so on. It needs specific analysis in the light of the oilfield situation^17^. Sweep volume and oil displacement efficiency are two key aspects influencing oil recovery efficiency, and they are sensitive to a variety of static geological factors, such as wettability^18–20^, permeability^21–23^, pore throat structure^24–26^, and other reservoir physical properties.

Since its introduction into the petroleum industry, nuclear magnetic resonance (NMR) technology has played an important role, where the relaxation mechanism is related to the hydrogen atoms in the formation and can provide pores and fluids information unrelated to formation lithology^27–29^. T_2_ spectrum can be used to accurately calculate the reservoir’s total porosity and to study the quantitative distribution characteristics of average pore throat radius in rocks^30–32^. In recent years, NMR fluid property identification technology and NMR imaging technology have advanced rapidly. The fluid properties in the pore throat can be accurately identified through further construction and decomposition of the T_2_ spectrum. At the same time, the signal is spatially located and further processed by a linear gradient magnetic field to form a clear color image that accurately reflects the distribution of different fluids within the sample, providing a new method for qualitative observation and quantitative research of the remaining oil distribution^33–36^.

Previous studies made in-depth analysis on chemical flooding in light of EOR mechanism^37–39^ and system^40,41^. Reservoir physical properties also represent an important factor controlling the chemical flooding recovery. Some scholars pointed out that such reservoir physical properties as capillary number^42^, permeability^43^ and heterogeneity^44–46^ can control chemical flooding recovery. However, the influence of reservoir types on chemical flooding recovery has scarcely been discussed, and there is no quantitative visualization of remaining oil distribution. Moreover, the existing experimental researches on chemical flooding are mainly aimed at EOR, but seldom simulate the oilfield development process considering the economic benefits. Therefore, these researches cannot provide direct and effective theoretical guidance for development of, especially, high water-cut oilfields.

In this paper, the NMR imaging displacement experiments of waterflooding combined with polymer flooding and polymer-surfactant flooding are performed on core samples with different reservoir physical properties taken from an ultrahigh water-cut oilfield in western China depending upon the reservoir geology and development parameters, to simulate the oilfield development process considering the economic benefits. The displacement stage with the best oil displacement effect in the process of waterflooding, chemical flooding and then waterflooding was defined, and the control effect of permeability and heterogeneity on the improvement of oil displacement efficiency by polymer-surfactant binary flooding was discussed. The distribution position of remaining oil in different displacement stages was quantitatively and visually displayed, and its control factors were revealed. The study results provide theoretical guidance for tapping the remaining oil potential of old oilfields with high and ultrahigh permeabilities.

## Experimental methods

### Experimental materials and equipment

#### Core samples

Three groups of high and ultrahigh permeability sandstone core samples were chosen from an ultrahigh water-cut oilfield in western China, and they were all drilled along layers from cores and named sample 1, sample 2, and sample 3, respectively. Each group contains two cylindrical cores, one was 2.5 cm in diameter and 10 cm in length and the other was a parallel sample of the first, 2.5 cm in diameter and 8 cm in length. Each group of 10-cm cylindrical cores was cut into two sections of approximately 5-cm length for the NMR imaging displacement experiment. Each of the 8-cm cylindrical cores was cut into three sections with 4-cm, 2.5-cm and 1.5-cm length, respectively. Wettability test, high-pressure mercury injection test and SEM imaging displacement experiment were performed on these samples to analyze the reservoir physical properties, pore throat morphology and mineral occurrence. Wettability was measured by self-imbibition method. The ratio of water spontaneously inhaled by the core to the total water displacement and the ratio of oil spontaneously inhaled by the core to the total oil displacement were compared to determine the relative wetting index and judge the wettability. According to the injection pressure and the amount of mercury injected, the capillary pressure curve was plotted, and the average pore throat radius and homogeneity coefficient, which can reflect the pore throat size and distribution, were calculated. The above experiments were carried out at the China University of Petroleum (East China) Key Laboratory of Deep Oil and Gas and the Shengli Oilfield Exploration and Development Research Institute’s Petroleum Geology Test Center. Table 1 displays some sample data obtained via high-pressure mercury injection and wettability testing.

**Table 1:** Partial reservoir parameters of experimental samples.

Sample	Porosity /%	Permeability /mD	Homogeneity coefficient	Average pore throat radius /μm	Relative wetting index	Wetting category
1	34.4	5210	0.401	14.11	0.27	Weak hydrophilicity
2	34.4	2280	0.459	10.42	0.28	Weak hydrophilicity
3	29.3	886	0.325	9.787	0.12	Weak hydrophilicity

#### Experimental fluid

Due to the presence of hydrogen protons, both water and hydrocarbons can generate NMR signals^47–49^. To distinguish hydrocarbons and water in the pore throat, the oil used in the NMR imaging displacement experiment is perfluorinated oil, which does not produce nuclear magnetic signals, can separate oil from water and chemical signals, and has a viscosity of 8.42 mPa·s and a density of 1.8 g/cm^3^. The experimental water has a total salinity of 4863 mg/L, a binary oil displacement agent of 2000 mg/L polymer + 2500 mg/L surfactant, which IFT is 0.0076 mN/m. The concentration and the viscosity of the polymer is 2000 mg/L and 20.5 mPa s, respectively. The polymer is anionic polyacrylamide, and the surfactant is petroleum sulfonate. To make the results more accurate, the reservoir temperature, which is 58 °C, was consulted, and all experimental fluid properties were measured at 58 °C.

#### Experimental equipment

**Figure 1:**
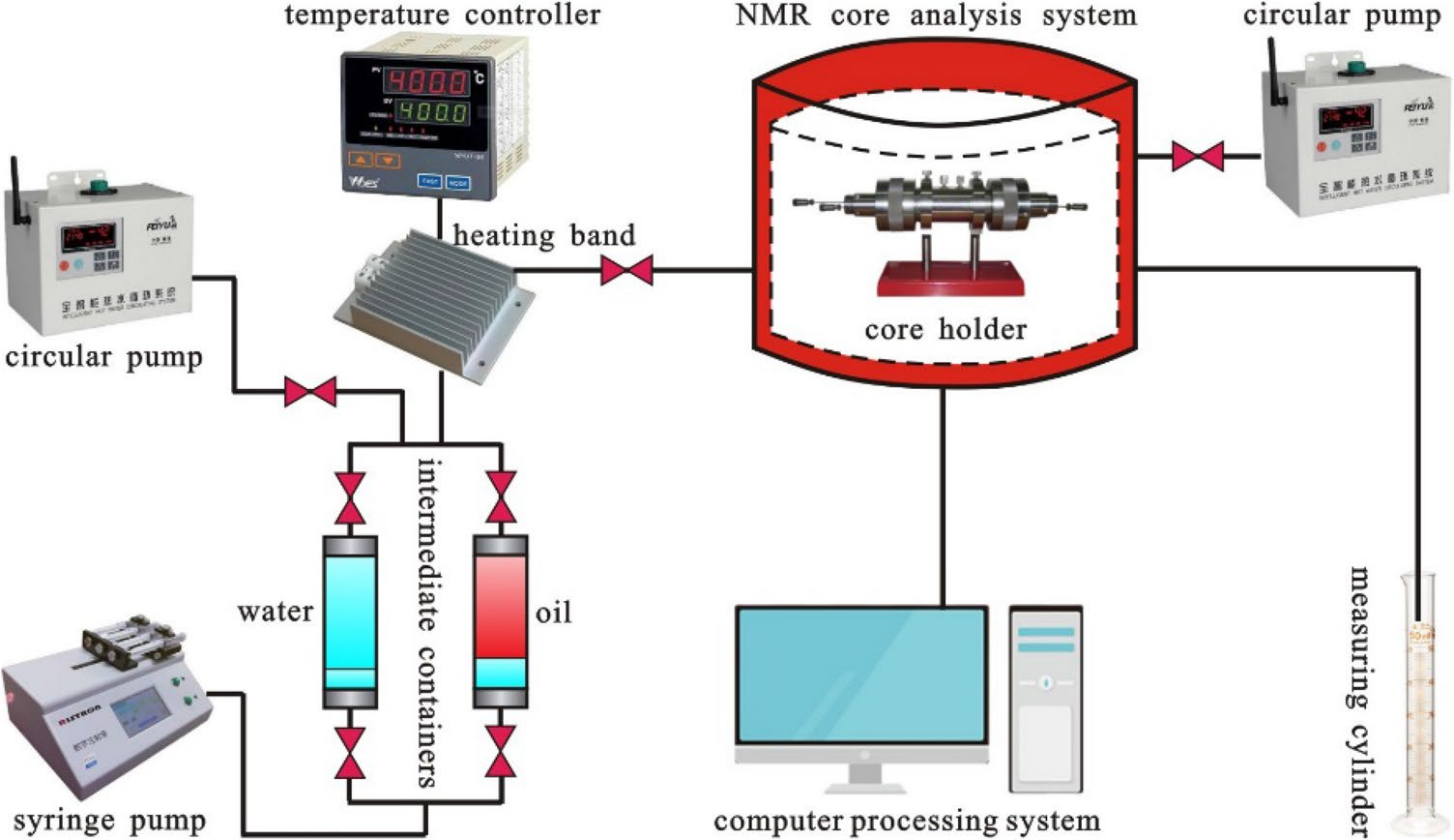
Schematic diagram of core displacement system for nuclear magnetic resonance imaging.

The MacroMR12-110H-1 NMR imaging core displacement system was used in the NMR imaging displacement experiment. This system primarily consists of an NMR core analysis system, a computer processing system, a circular pump, a syringe pump, a temperature controller, a heating band, a measuring cylinder, and intermediate containers (Fig. 1). The NMR T_2_ spectrum for the samples is obtained using the NMR core analysis system, and the Carr–Purcell–Meibom–Gill sequence is chosen. Set the waiting time to 6 s, the ECHO interval to 0.12 ms, the echo number to 18,000, and the scan times to 32. The experiment was carried out in accordance with the Specification for Measurement of Rock NMR Parameter in Laboratory (SY/T 6490-2014). The wettability test was carried out using a steady-state phase-permeation displacement system and an YXL-15 ultrahigh speed centrifuge, in accordance with the Test Method of Reservoir Rock Wettability (SY/T 5153-2007). The instrument used for high-pressure mercury intrusion is a high-performance automatic mercury intrusion instrument (AutoporeIV9500 S/N1324), in accordance with the Rock Capillary Pressure Measurement (GB/T 29171-2012). The instrument used for scanning electron microscope is a Quanta 200 scanning electron microscope, in accordance with the Analytical Method of Rock Sample by Scanning Electron Microscope (SY/T 5162-2014).

### NMR imaging displacement experimental scheme

**Figure 2:**
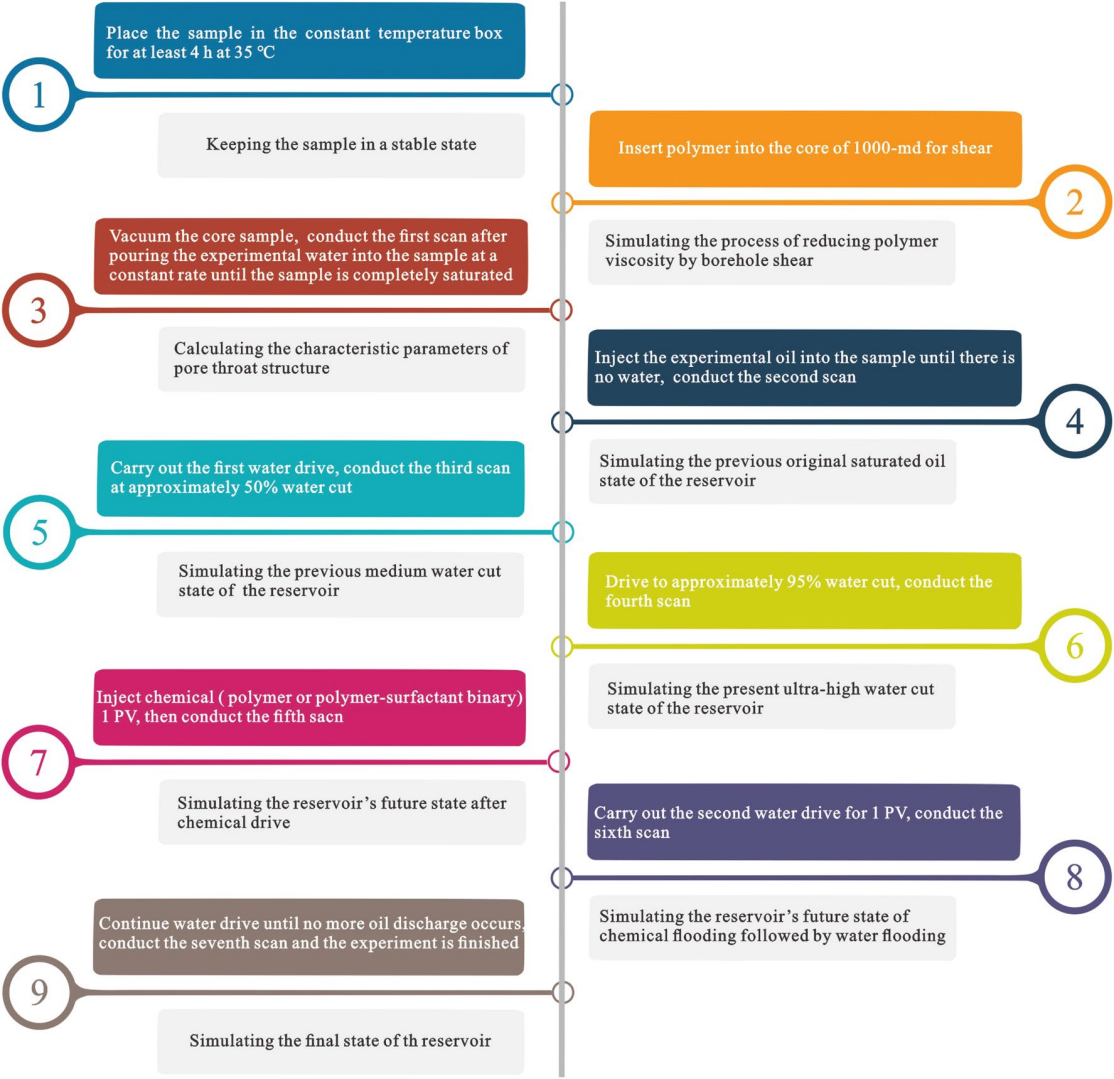
Flowchart of the NMR imaging displacement experiment.

Previous studies indicate that the oil recovery and economic benefits of high water-cut oilfields can be maximized by changing waterflooding to tertiary oil recovery at the proper time^50^. Here, the oilfield development process, i.e., waterflooding → chemical flooding → waterflooding, was simulated. Six groups of experiments were designed to compare the remaining oil distribution and oil recovery by chemical flooding in cores with different permeabilities. The displacement rate remained constant in the experiments. According to the effect time of isotopic tracer, the displacement rate of coring well group is 5 m/day on average, while the displacement rate in experiments is set at 0.35 mL/min after conversion to the core. Table 2 shows the experimental conditions and schemes. The experimental procedure is the same, including nine steps (Fig. 2):

**Table 2:** NMR imaging displacement experiments.

Experiment Number	Sample	Experimental temperature/°C	Experimental pressure	Chemical agent
1	1	58 (the reservoir temperature)	Atmospheric pressure	Polymer
2				Polymer + surfactant
3	2			Polymer
4				Polymer + surfactant
5	3			Polymer
6				Polymer + surfactant

### Acquisition of displacement experimental results by NMR imaging

Following the completion of the experiment, the T_2_ spectrum and NMR oil-bearing images can be obtained using the experimental data and image processing system. The injection multiple as well as the volume of produced oil and water can be directly measured during the experiment. According to the T_2_ spectrum, the oil saturation at various stages of displacement can be calculated:

1 $$Soi={100\%-S}_{w}\cdot {\int }_{0}^{{T}_{2\mathrm{max}}}m{\left({T}_{2i}\right)}_{{S}_{oi}}d{t}_{2} /{\int }_{0}^{{T}_{2\mathrm{max}}}m{\left({T}_{2i}\right)}_{100\%}d{t}_{2}\times 100\mathrm{\%}$$

Oil displacement efficiency in each stage:

2 $$\eta =\left({S}_{o}-{S}_{or}\right)/{S}_{o}\times 100\mathrm{\%}$$

where *S*_*w*_ is the initial water saturation, %; *S*_*o*_ is the initial oil saturation, %; ${S}_{or}$ is the remaining oil saturation, %; *T*_2max_ denotes the maximum T_2_ time, ms; $m{\left({T}_{2i}\right)}_{{S}_{oi}}$ is the T_2_ spectrum amplitude after displacement, a.u.; $m{\left({T}_{2i}\right)}_{100\%}$ is the T_2_ spectrum amplitude for the fully oil-saturated condition, a.u.

The pore throat radius can be converted by T_2_ relaxation time^49,51^.

3 $$\frac{1}{{T}_{2}}=\frac{1}{{T}_{2b}}+\frac{1}{{T}_{2s}}+\frac{1}{{T}_{2d}}$$

where ${T}_{2b}$ is the transverse relaxation time of the pore fluid in a sufficiently large vessel, ms; ${T}_{2s}$ is the transverse relaxation time caused by surface relaxation, ms; ${T}_{2d}$ is the transverse relaxation time of pore fluid caused by diffusion in a magnetic field gradient, ms.

Surface relaxation plays a significant role when a short recovery time is used, and the pores only contain saturated fluid, and ${T}_{2}$ is directly proportional to pore size:

4 $$\frac{1}{{T}_{2}}\approx \frac{1}{{T}_{2s}}={\rho }_{2}(\frac{S}{V})$$

where ${\rho }_{2}$ is the ${T}_{2}$ surface relaxation rate, μm /ms; $\frac{S}{V}$ is the specific surface area of pores, 1/μm.

S/V can be calculated as a function of a pore’s dimensionless shape factor, *F*_*s*_, and radius, r (μm):

5 $$\frac{S}{V}=\frac{{F}_{s}}{r}$$

Therefore, according to Formula (4) and Formula (5),

6 $$\frac{1}{{T}_{2}}={\rho }_{2}\frac{{F}_{s}}{r}$$

Define:

7 $$C={\rho }_{2}{F}_{s}$$

Then:

8 $${r=T}_{2} \cdot C$$

As the relaxation rate and pore shape factor Fs can be roughly regarded as constants for a specific core, coefficient C should also be a constant. After obtaining C, the NMR spectrum can be converted into the pore throat radius distribution. Scholars have proposed a number of NMR capillary curve construction methods based on mercury injection data calibration for calculating C value^52–54^. An improved similarity comparison method is used in this study. Accumulate from the largest pore on the right boundary to the smallest pore on the left boundary. Choose the pore throat radius range measured by mercury injection in the right accumulation curve as the comparable interval of NMR pore throat radius. Construct the spectral conversion pore throat radius distribution curve using the longitudinal interpolation method and the least square method, and find the best conversion coefficient C value between the ${T}_{2}$ value and the pore throat radius^52^. The conversion coefficients between T_2_ time and pore radius for samples 1, 2, and 3 are 0.093, 0.107, and 0.068 m/ms, respectively.

### Factors affecting the accuracy of experiment results

The following factors may affect the accuracy of experiment results:

1. The density of perfluorinated oil is 1.8 g/cm^3^, which is much higher than that of crude oil and water.
2. The interaction of perfluorinated oil with water and chemical agent is different from that of crude oil with water and chemical agent.
3. In the process of displacement, the experiment must be interrupted for NMR detection, which has an effect on the measurement of oil and water and the distribution of oil and water in the core.

## Results and analysis

### Reservoir physical property characteristics

**Figure 3:**
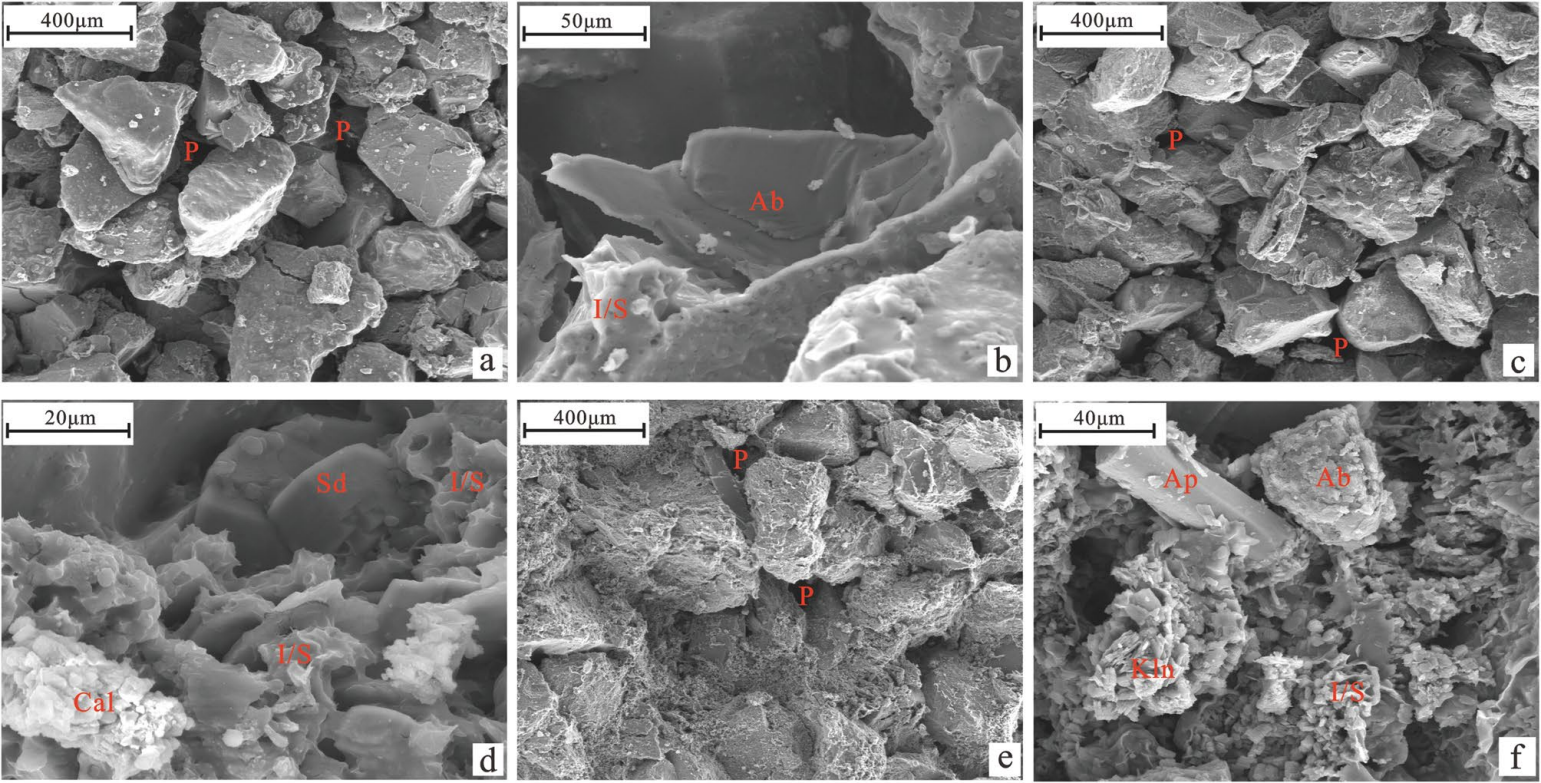
Microscopic reservoir characteristics of experimental samples (*P* pore, *Ab* albite, *I/S* illite-montmorillonite mixed layer, *Sd* siderite, *Cal* calcite, *Ap* apatite, *Kln* kaolinite).

**Figure 4:**
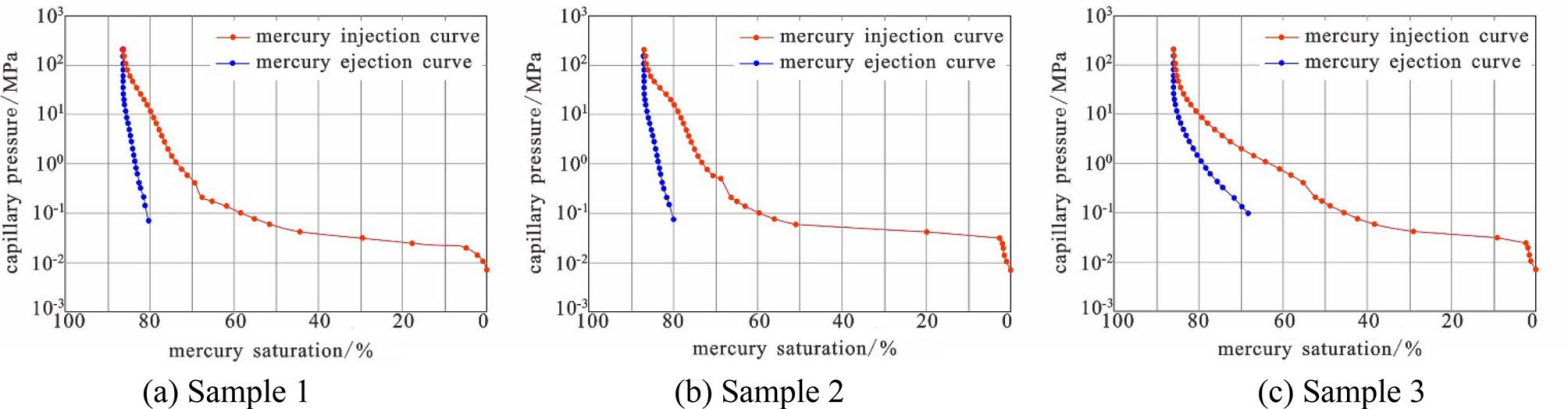
High pressure mercury injection curve of experimental sample.

As can be seen from Table 1, the wettability of the three samples after long-term water injection development is weak hydrophilic. Therefore, the difference in reservoir physical properties between the three samples in this study is primarily reflected in differences in microscopic pore throat structure and permeability. The reservoir physical properties of the three samples differ significantly, according to the classification of porosity and permeability of clastic reservoirs in the Evaluating Methods of Oil and Gas Reservoirs (SY/T6285-2011) and the results of the scanning electron microscopy and high-pressure mercury injection analysis. Sample 1 has the highest permeability; it has ultrahigh porosity and ultrahigh permeability, has good sorting, a smooth particle surface, well-developed primary pores (Fig. 3a), and good pore connectivity. Sample 1 exhibits a dissolution phenomenon that exists in albite particles and the mixed layer filled between particles (Fig. 3b), forming secondary pores, a long gentle section of mercury inlet curve, and a low displacement pressure (Fig. 4a). Sample 2 has medium permeability and is an ultrahigh porosity and ultrahigh permeability reservoir with the best sorting, smooth particle surface, mainly primary pores, and acceptable pore connectivity (Fig. 3c). Calcite, flaky mixed layer, and siderite are filled between particles; calcite can be dissolved (Fig. 3d). The gentle section of the mercury inlet curve is longer, and the displacement pressure is small (Fig. 4b). Sample 3 has low permeability, is a high porosity and high permeability reservoir, and is sorted with relatively few primary pores and poor pore connectivity (Fig. 3e). Furthermore, the intergranular pores are filled with apatite, hexagonal kaolinite, flaky illite mixed layer, etc. (Fig. 3f). The gentle section of the mercury inflow curve is short and not obvious, and the displacement pressure is high (Fig. 4c).

**Figure 5:**
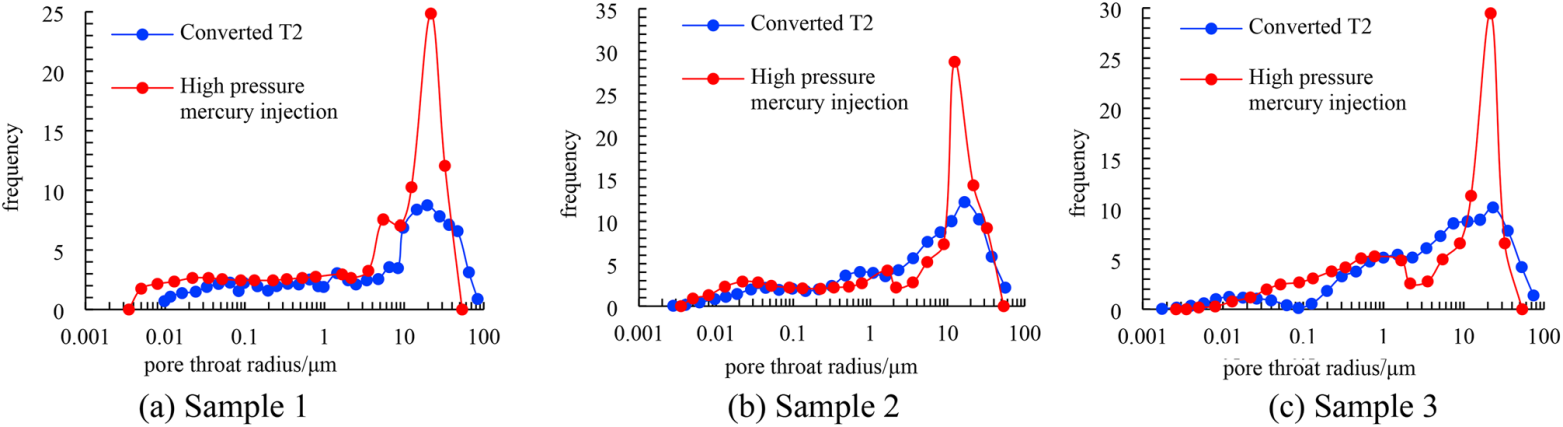
Pore-throat distributions obtained with high pressure mercury injection, and the converted pore-throat sizes obtained from the water saturated T_2_ spectra for samples.

A pore throat radius distribution frequency diagram obtained through spectral conversion and high-pressure mercury injection is shown in Fig. 5. The morphological characteristics of the pore throat radius distribution frequency diagram obtained by the two methods are essentially similar for each sample, demonstrating that the conversion coefficient C is relatively accurate. When the distribution frequencies of the three samples are compared, it is also clear that the pore throat radius distribution of sample 2 is the most concentrated, the pore throat radius distribution of sample 3 is the most dispersed, and sample 1 is in the middle, corresponding to the homogenization coefficient in Table 1 and the gentle section characteristics of the tribute curve in Fig. 4.

### T_2_ spectral characteristics of different displacement stages

**Figure 6:**
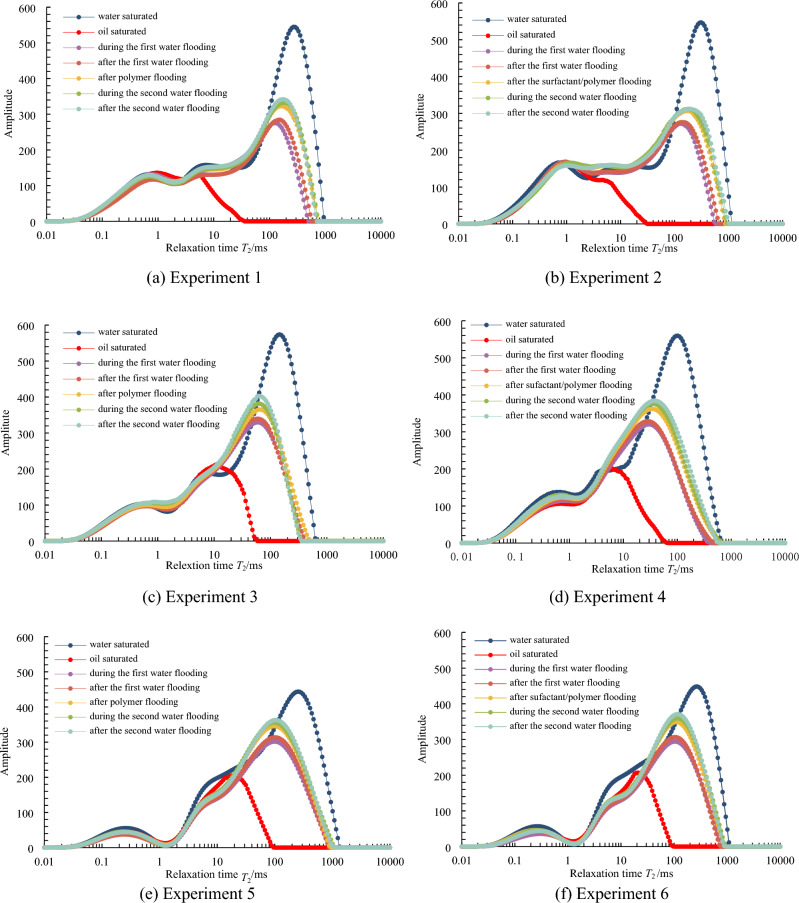
T_2_ spectra during different displacement periods for the 6 experiments.

Figure 6 depicts the spectra of six experiments. Using the spectrum from experiment 1 as an example (Fig. 6a), the saturated oil spectrum is bimodal, with short relaxation time peaks distributed between 0.61 and 1.6 ms (or with pore throat radius of 0.06–0.15 μm) and long relaxation time peaks distributed in the range of 3.6–6.1 ms (0.33–0.57 μm). Short relaxation time peaks are distributed in the range of 0.43–1.6 ms (0.04–0.15 μm), intermediate time peaks are distributed in the range of 4.2–8.0 ms (0.39–0.74 μm), and long relaxation time peaks are distributed in 98–290 ms (9.11–26.97 μm). As the NMR signal cannot be measured in the experimental oil, the signal simulating formation water and polymer is detected. As a result, as the displacement increases, the amplitude of the spectral signal increases, indicating that water and chemicals are injected, and the oil in the pore throat is displaced and discharged. The amplitude of the spectral signal increases the most from the saturated oil stage to displacement to 50% water cut, and the second-largest increase is from the end of the first waterflooding to the end of polymer flooding, as shown by the change in spectral signal amplitude in different displacement stages. This stage demonstrates that most oil is flooded in these two stages. Moreover, the signal variation amplitude in each stage is the greatest after the relaxation time is greater than 90 ms (8.37 μm), indicating that the oil is primarily discharged from the pore throat after the relaxation time is greater than 90 ms (8.37 μm). The characteristics of other samples’ single spectra are essentially the same as those of sample 1.

When the spectra of different chemical agents in the same sample are compared, it is discovered that the spectrum increase of polymer-surfactant binary flooding is greater than that of polymer flooding from the end of primary waterflooding to the end of chemical flooding, indicating that polymer-surfactant binary flooding has a better oil displacement effect than polymer flooding. This is most noticeable in the second group (Fig. 6c,d).

When the spectra of different samples flooded by the same chemical agent are compared, it is discovered that from saturated oil to the end of the second waterflooding, the variation range of the spectrum decreases with the decrease of reservoir permeability (Fig. 6b,d,f), indicating that the lower the permeability, the less oil is flooded.

### Influence of reservoir physical properties and chemical agents on oil displacement efficiency

**Figure 7:**
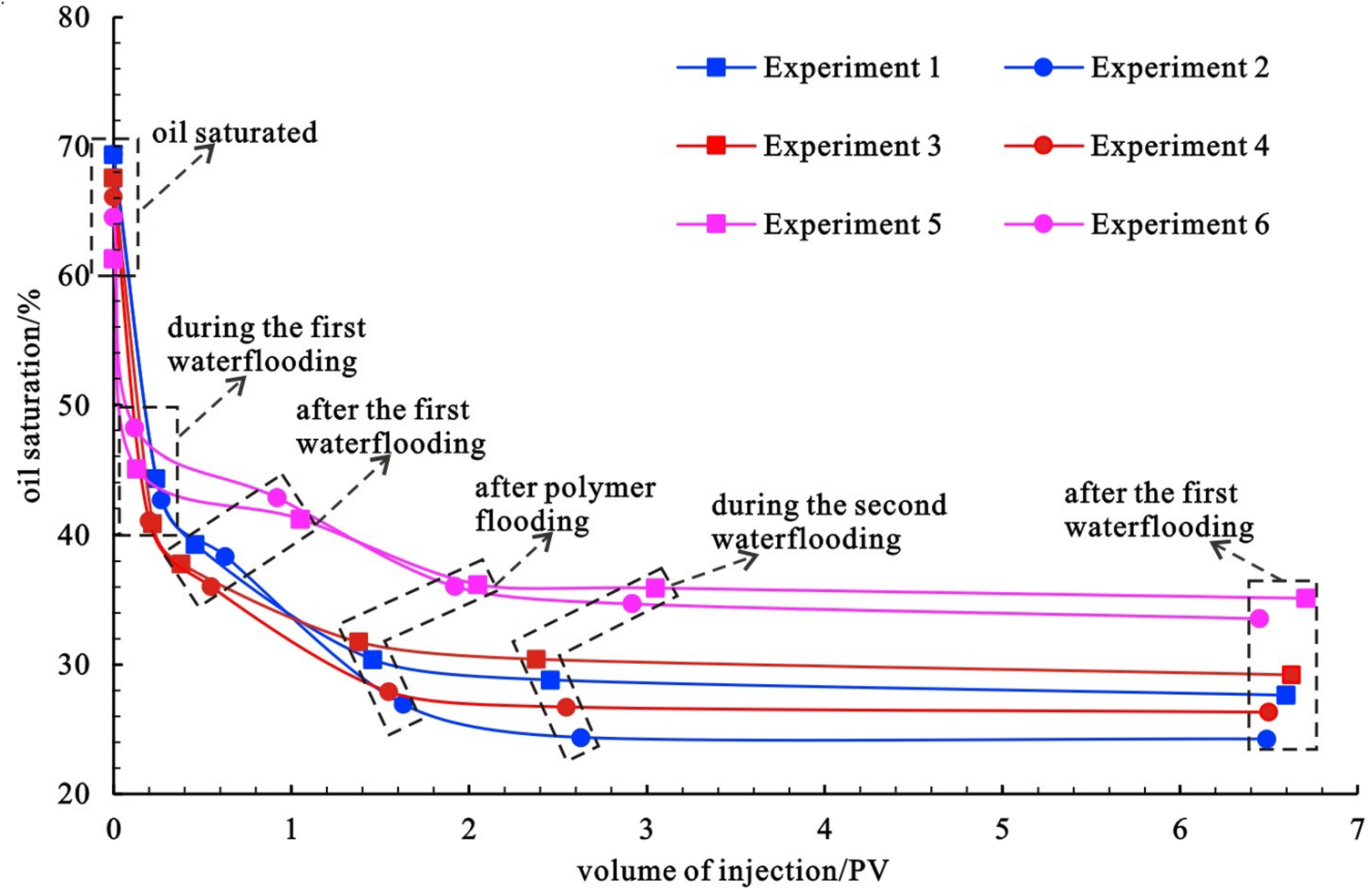
Variation relationship of oil saturation displacement multiple in 6 groups of experiments.

**Figure 8:**
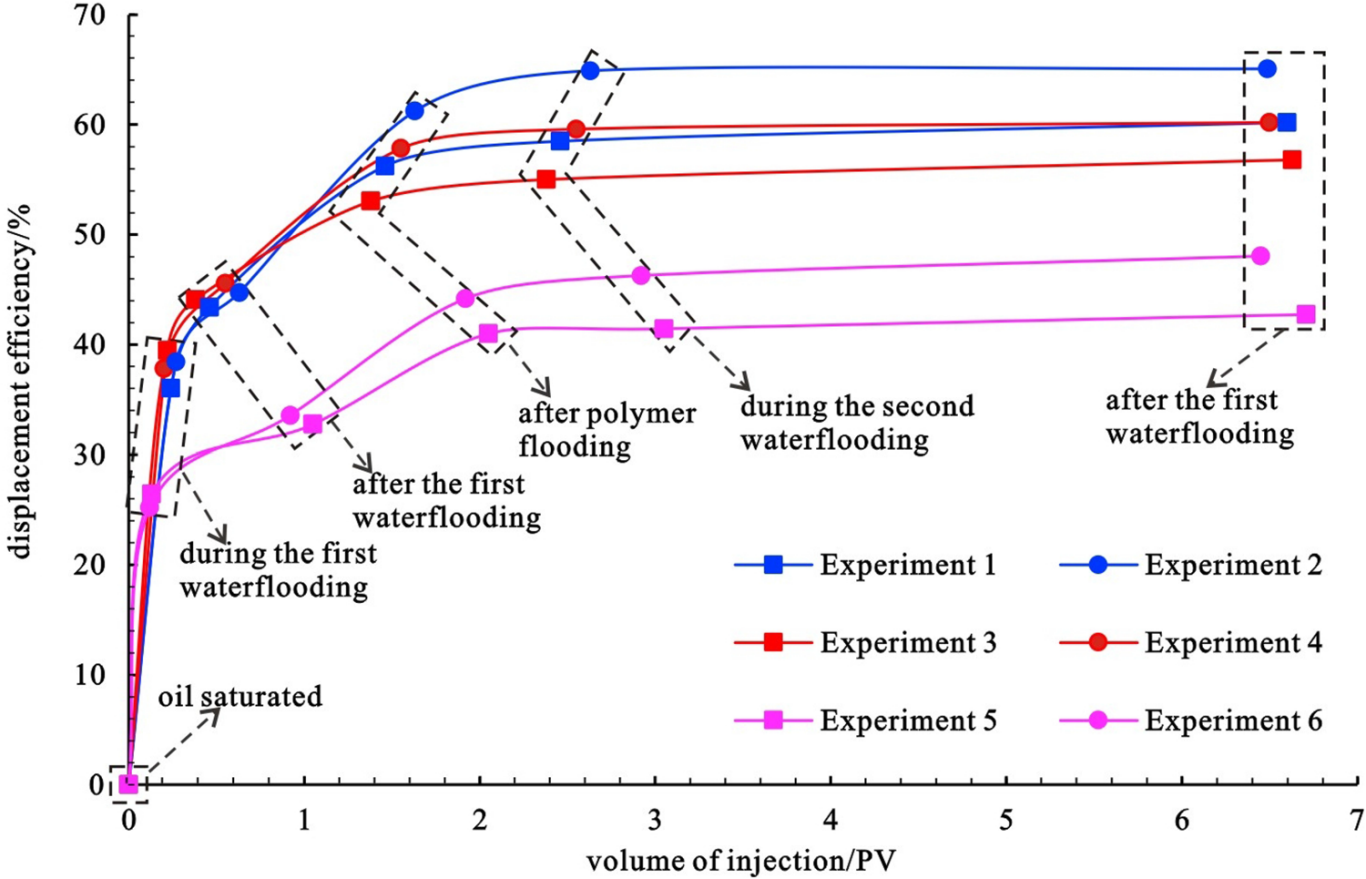
Relationship between oil displacement efficiency and displacement multiple in 6 groups of experiments.

The relationship between oil saturation and oil displacement efficiency with displacement multiple is recorded in each group of experiments to investigate the influence of reservoir physical properties on the oil displacement efficiency of waterflooding–chemical flooding (Figs. 7 and 8). Table 3 displays the key data from each group of experiments. In each experiment, the cumulative injection of chemical agents is the same, all of which are 1 PV, and the cumulative injection of water is essentially the same, about 5.5 PV. Experiment 2 exhibits the highest final oil displacement efficiency of 65.1%, whereas experiment 5 exhibits the lowest final oil displacement efficiency of 42.7%. Comparing the experiments of different samples with the same chemical agent, the final oil displacement efficiency gradually decreases with the deterioration of reservoir physical properties from experiment 1 to experiment 3 to experiment 5, with a difference of 17.5%. From experiment 2 to experiment 4 to experiment 6, the final oil displacement efficiency gradually decreases with the deterioration of reservoir physical properties, with a difference of 17.1%. When the physical properties of the samples used in experiment 1 and experiment 2 are compared, the physical properties of the samples used in experiment 1 and experiment 2 are the best, and the final oil displacement efficiency of experiment 2 of polymer-surfactant binary flooding is 4.9% higher than that of experiment 1 of polymer flooding only. The physical properties of the samples used in experiments 3 and 4 are medium, and the final oil displacement efficiency of experiment 4 of polymer-surfactant binary flooding is 3.4% greater than that of experiment 3 of polymer flooding alone. The physical properties of the samples used in experiments 5 and 6 are poor, and the final oil displacement efficiency of experiment 6 of polymer-surfactant binary flooding is 5.3% greater than that of experiment 5 of polymer flooding alone. The results of the preceding analysis show that increasing permeability and adding surfactant based on the polymer can help to improve the final oil displacement efficiency.

**Table 3:** Key parameters of 6 groups of experiments.

Experiment Number	Saturated oil saturation/%	Final oil saturation /%	Final displacement efficiency/%	Cumulative injected water /PV	Cumulative injection of chemicals /PV
1	69.4	27.6	60.2	5.60	1.0
2	69.4	24.2	65.1	5.49	1.0
3	67.6	29.2	56.8	5.63	1.0
4	66.1	26.3	60.2	5.50	1.0
5	61.3	35.1	42.7	5.71	1.0
6	64.5	33.6	48.0	5.45	1.0

As shown in Figs. 7 and 8, as the displacement multiple increases, whether it is oil saturation or oil displacement efficiency, the two sections of the curve with the greatest slope are from saturated oil displacement to 50% water cut and from the end of the first waterflooding to the end of polymer flooding, indicating that oil saturation decreases and oil displacement efficiency increases in these two stages. The speed is the fastest, which is consistent with the results of the spectral analysis.

### Influence of reservoir physical properties and chemical agents on remaining oil distribution

**Figure 9:**
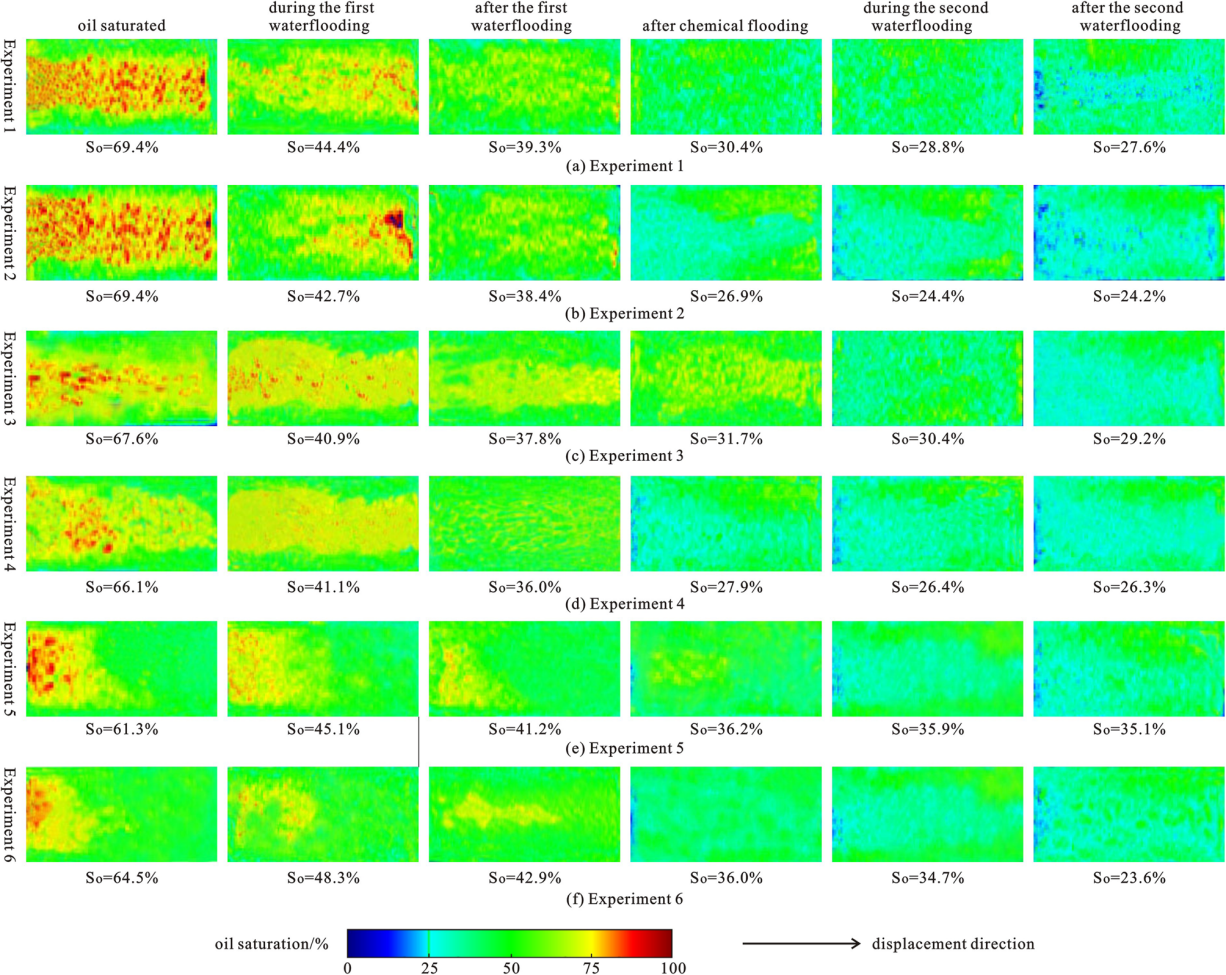
Distribution image of remaining oil in different displacement stages of NMR flooding in 6 groups of experiments.

NMR imaging can be used to observe and analyze the remaining oil distribution at various displacement stages. Figure 9 depicts the remaining oil distribution of NMR imaging from six experiments at various stages. Experiment 1 and 2 samples have the highest permeability and sorting ratio. According to Fig. 9a,b, when the oil is saturated in Experiment 1 and Experiment 2, the oil saturation is the highest, and the oil distribution is relatively uniform in the bedding direction. When the water cut is 50%, the oil saturation clearly decreases overall, with an average decrease of 25.9%, and it decreases further at the inlet section. The remaining oil is mostly concentrated in the middle and the outlet sections. When the water cut is set to 95%, the oil saturation drops by 4.7% on average, and the total water injection is about 0.5 PV. The remaining oil is distributed evenly after further waterflooding. The oil saturation of experiment 2 using polymer-surfactant binary flooding is reduced by 11.5% in the 1 PV chemical flooding stage, while the oil saturation of experiment 1 using polymer flooding is reduced by 8.9%. Experiment 2 outperforms experiment 1 by 2.6%. The remaining oil distribution in experiment 1 is still relatively uniform because the sample is relatively homogeneous, and the polymer has the function of plugging the high permeability layer and reducing flow fingering of the displacement fluid to improve sweep efficiency^55–57^. Surfactants can reduce the interfacial tension between oil and water^56^ in experiment 2, making it easier to displace and discharge the remaining oil. Due to fingering phenomena caused by gravity and heterogeneity^36^, the oil saturation is lower at the inlet and lower part of the sample, and the remaining oil is mainly distributed at the outlet and top of the sample. The oil saturation decreased further during the second waterflooding process. In experiment 1, the oil saturation decreased more from 1 PV of the second waterflooding to the end of the experiment, forming a more obvious low-value area of oil saturation in the middle of the sample. Experiment 2 saw the remaining oil discharged more evenly, which occurs mostly at the top near the outlet section.

The permeability of the samples used in experiments 3 and 4 is medium, but they are the most homogeneous. The remaining oil distribution characteristics of experiment 3 and experiment 1, as well as those of experiment 4 and experiment 2 are very similar, as shown in Fig. 9a–d. Furthermore, the oil saturation of experiment 4 using polymer-surfactant binary flooding is reduced by 8.1% in the 1 PV chemical flooding stage, whereas the oil saturation of experiment 3 using polymer flooding is reduced by 6.1%. Experiment 4 yields a 2% lower result than experiment 3. As the samples used are more homogeneous, the remaining oil distribution in each stage of experiments 3 and 4 is more uniform, especially after the displacement to 50% water cut and the end of the experiment, this phenomenon is more obvious, and the oil saturation at the inlet and the outlet sections is closer.

Experiments 5 and 6 used samples with the lowest permeability and the greatest heterogeneity, respectively. According to Fig. 9e,f, when saturated with oil, the oil saturation in experiments 5 and 6 is the lowest, and it is primarily distributed at the inlet section. The oil saturation at the inlet section decreases noticeably as the experiment progresses. Owing to the high heterogeneity, oil saturation in the middle section rises after the first water drive. The oil saturation of experiment 6 using polymer-surfactant binary flooding decreased by 6.9% in the stage of 1 PV chemical flooding, the oil saturation of experiment 3 using polymer flooding decreased by 5%, and experiment 6 decreased by 1.9% more than experiment 5. During the second waterflooding, fingering becomes more visible, the oil saturation in the middle decreases, and the remaining oil is primarily distributed at the top and bottom.

By comparing the six groups of experiments, it is clear that reservoir permeability and displacement media determine overall oil saturation and oil displacement efficiency, whereas reservoir heterogeneity and gravity control the distribution position of the remaining oil. The higher the heterogeneity, the more visible is the fingering phenomenon. The remaining oil is mainly distributed at the outlet end and the top of the sample. The oil saturation reduction during chemical flooding in Experiments 1 and 2, Experiments 3 and 4, and Experiments 5 and 6 shows that polymer-surfactant flooding has a better oil displacement effect than polymer flooding, and the higher the permeability of reservoirs, the better the effect of displacing remaining oil and improving oil displacement efficiency. Combined with the reservoir physical properties of the experimental samples, it can be concluded that permeability plays a more obvious controlling role in improving the oil displacement efficiency of polymer-surfactant binary flooding compared with heterogeneity. On the one hand, the higher the permeability of the reservoir, the better the effect of the polymer to increase the conformance efficiency, and the surfactant can play a fuller role in reducing the interfacial tension, and better overcome the surfactant retention. On the other hand, due to the common influence of many factors, heterogeneity does not show obvious correlation to improve the oil displacement efficiency of polymer-surfactant binary flooding.

## Summary and conclusions

1. The first waterflooding in the low water-cut stage and chemical flooding in the ultrahigh water-cut stage have the best oil displacement effect. ① During the two stages, one is from saturated oil flooding to 50% water cut and the other one is from 95% water cut to the end of 1 PV polymer flooding, the T_2_ spectral signal amplitude increases the most, the oil saturation decreases the most, and the oil displacement efficiency increases the most when simulating oilfield waterflooding, followed by chemical flooding, and then waterflooding again. ② The oil is primarily discharged from the pore throat larger than 90 ms (8.37 μm).
2. Compared with heterogeneity, permeability plays a more obvious controlling role in improving the oil displacement efficiency of polymer-surfactant binary flooding: ① The higher the permeability of the reservoir, the better the effect of the polymer to increase the conformance efficiency, and the surfactant can play a fuller role in reducing the interfacial tension, and better overcome the surfactant retention. ② Due to the common influence of many factors, heterogeneity does not show obvious correlation to improve the oil displacement efficiency of polymer-surfactant binary flooding.
3. The influence of fingering phenomenon on the distribution of remaining oil is most obvious in the second waterflooding, and the distribution of remaining oil with polymer slug is more obviously affected by the fingering phenomenon than that with polymer-surfactant slug. Due to the joint action of fingering phenomenon and gravity, the remaining oil is mainly distributed at the outlet and the top of the sample.

## Data Availability

The datasets used and analysed during the current study available from the corresponding author on reasonable request.
